# Relationships between male secondary sexual traits, physiological state and offspring viability in the three-spined stickleback

**DOI:** 10.1186/s12862-021-01958-8

**Published:** 2022-01-07

**Authors:** Violette Chiara, Alberto Velando, Sin-Yeon Kim

**Affiliations:** Grupo Ecoloxía Animal, Torre CACTI, Centro de Investigación Mariña, Campus de Vigo, Universidade de Vigo, 36310 Vigo, Spain

**Keywords:** Courtship, Genetic quality, Good-genes, Life history, Oxidative damage, Stickleback, Sexual selection

## Abstract

**Background:**

Sexual signals produced by males play a central role in sexual selection, but the relationship between these traits and the quality of the bearer are often ambiguous. Secondary sexual traits may represent genetic quality of the bearer, resulting in positive relationships with physiological state, or may be costly to produce, showing trade-off with physiological state. A number of studies have explored the relationships between secondary sexual traits and other functional traits, but few have studied their fitness consequences. We studied the link between diverse physiological traits and both morphological and behavioural sexual traits and examined how their interplay influences offspring viability in the three-spined stickleback.

**Results:**

Male sticklebacks showing nest building and courtship behaviour were smaller than those not investing in reproductive activities. There was no evidence that the expression of red nuptial colouration and the quality of courtship behaviour of males are positively related to their metabolic rates, swim ability, oxidative damage and mtDNA copy number. However, individuals showing larger red nuptial colour areas had higher levels of oxidative DNA damage in their sperm. Male courtship behaviour and aggressiveness, but not red colour area, were good predictors of offspring hatching and survival.

**Conclusions:**

Our results suggest that, in our study population at the southern edge of the species’ distribution, sexual colouration of male sticklebacks was not a good indicator of their body state, but both courtship quality and aggressiveness during the courtship are reliable cues of their gamete quality, influencing the viability of their offspring. Thus, females that choose mates based on their courtship behaviour will have high fitness. In the study population, which represents a fast pace-of-life with high reproductive rate and short lifespan, sexual ornaments of males may not honestly signal their physiological and physical state because they invest at maximum in a single reproductive season despite high costs.

**Supplementary Information:**

The online version contains supplementary material available at 10.1186/s12862-021-01958-8.

## Background

The presence and maintenance of elaborated secondary sexual traits has been commonly linked with direct or indirect fitness advantages for choosy individuals, typically females [1]. In some cases, secondary sexual traits and female preference can evolve through direct benefits if, for example, ornament size and colour reflect the state of the bearer [2]. Indeed, large or colourful ornaments of males are often associated with good internal state related to adequate alimentation [3–6], absence of parasitism or disease, or better immune response [7–11], thereby enhancing offspring viability through parental care [12, 13] (but see [14]). According to the “good genes” and “sexy sons” hypotheses, choosy females may also gain indirect fitness benefits through better genetic quality and attractiveness of their offspring (see [15, 16]). However, the relationships among sexual traits, physiological condition and genetic quality of males are often different across studies, and it is still unclear how the interplay between these male qualities influences their offspring [17].

Some studies have explained variation among males in secondary sexual traits using the concept of a fast-slow life-history continuum. Fast-living individuals invest more in sexual ornaments or weapons and benefit from increased mating success or higher dominance status [18] but have shorter life span than slow-living individuals that invest less in these sexual traits [19–21]. This is because the expression and maintenance of sexual traits are costly and divert resources away from other functional traits of the bearers, including somatic maintenance [16, 19]. These trade-offs between sexual traits and internal states may be mediated by energy expenditure and metabolism (see [22] for a review). The maintenance of sexual characters may require increased energy metabolism. For instance, resting metabolic rate is positively correlated with prenuptial gland size in male bank voles [23] and with sexual colouration in male and female American goldfinches [24]. One way to meet energetic demands of expressing secondary sexual traits may be increasing mitochondrial DNA (mtDNA) copy number in somatic cells to enhance cellular energy production. In fishes, reduced mtDNA copy number is associated with a dysfunction of mitochondrial activity in aged [25, 26] and larva fish [27], but it is unknown whether mitochondrial function can also influence the expression of sexual traits.

Long-term expression and maintenance of energetically costly sexual traits may result in an increased risk of oxidative damages. Normal metabolic processes generate reactive by-products, which induce oxidative damages in DNA, proteins and lipids [28, 29]. Some empirical studies have shown that males investing heavily in secondary sexual traits not only suffer accelerated somatic deterioration [30] but also fail to maintain germ cells free of damage [29, 31]. Deleterious oxidative damages in the soma reduce the individual’s lifespan and future reproduction opportunities [32], and those in the germline can affect the immediate reproductive success by reducing fertility and offspring viability [29, 33–36].

Courtship displays are energetically costly secondary sexual traits. Metabolic rate can increase up to four-fold during courtship in the wax moth [37] and up to three-fold during singing in the Carolina wren [38] (for a review, see [39]). Although the energetic cost of a single courtship event may be almost negligible in comparison to the daily energy budget of an individual [39], there is evidence of long-term energetic costs and time costs of courtship in various species [40, 41]. Thus, the quality and intensity of courtship display of a male may honestly signal its physiological condition or genetic quality. Indeed, in Sierra dome spiders the quality of energetically costly courtship is related to the male’s metabolic rate [42]. In golden-collared manakins, females seem to select their mate on the basis of metabolic competence and courtship quality of males is related to their heart rate [43]. Thus, physical performances and maximum metabolic rate of a male may predict its courtship quality [39]. In species in which males defend a territory and build a nest, the quality of nest or territory may also represent time and energy expenses of the male [41, 44]. Females can predict a male’s quality through its aggressiveness because this characteristic is related with the ability to protect offspring and to access resources in competitive environment [45, 46]. However, highly aggressive males may also harass females during courtship, decreasing their attractiveness (e.g., [47]). In fishes where males provide parental care, highly aggressive males should be avoided by females because high levels of aggression-inducing hormones (androgens) are related to reduced parental behaviours [48].

Here, we studied the link between physiological state and sexual signalling and examined how their interplay influences offspring viability in the three-spined stickleback (*Gasterosteus aculeatus*). We used a stickleback population at the southern edge of its distribution, which represents a fast pace-of-life with high reproductive rate and short lifespan. In this species, including our study population, males defend a territory, build a nest, provide paternal care, and express red nuptial colouration on their cheek and throat based on dietary carotenoids. This colouration is known to represent physical condition of males [49, 50] but incurs costs in terms of predation risk [51] and oxidative damage [29, 52]. Male sticklebacks perform highly stereotyped courtship displays, which represent their parenting qualities [13]. Males whose courtship was associated with aggressive behaviour are avoided by females [47]. In this study, we first studied whether different characteristics of the physiological state of male sticklebacks, including standard and maximum metabolic rates, swim ability, oxidative DNA damage in sperm, and mtDNA copy number in muscle, are related with their secondary sexual traits, such as nest, courtship behaviour, aggressiveness, and size of red nuptial colour area. Finally, we examined whether the physiological and sexual traits are good predictors of the offspring survival.

## Results

### Secondary sexual traits and physiological states

**Fig. 1:**
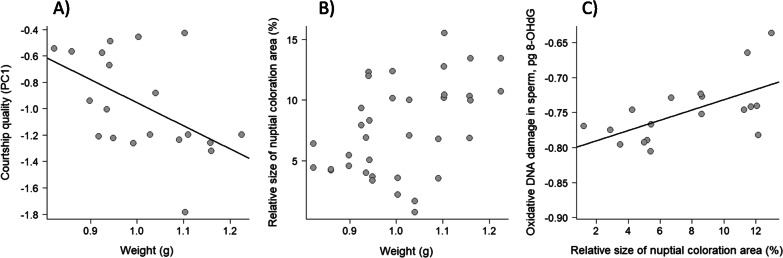
**A** Relationship between body weight and the quality of courtship in males with a nest (N = 20). **B** Relationship between body weight and relative size of red colour area (N = 36 measurements, N = 18 fish). **C** Relationship between relative size of nuptial colour area and the level of oxidative DNA damage in sperm (N = 18). Courtship and oxidative DNA damage in sperm are transformed values. Regression lines are presented for significant relationships (P < 0.05)

Among 29 males used in this study, 20 built a nest during the study period, and 18 of these succeeded to reproduce repeatedly. Fish with a nest were smaller in standard length than those without a nest (standard length: t = 2.964, df = 13.581, P = 0.011; Additional file 1: Table S1). Fish with and without a nest did not differ in residual standard metabolic rate (rSMR), residual maximum metabolic rate (rMMR), critical swimming speed (*U*_crit_), the level of oxidative DNA damage in sperm and mtDNA copy number in muscle (P ≥ 0.323; Additional file 1: Table S1). We assessed courtship quality and aggressiveness of all males that reproduced in a courtship behaviour assay. In these fish, courtship quality was negatively correlated with body weight (F_1;12_ = 6.498, P = 0.023, Fig. 1A), but heavier fish had a larger relative red area (F_1;12_ = 4.685, P = 0.051, Fig. 1B). Courtship quality, aggression and relative size of red colour area were not related to rSMR, rMMR, *U*_crit_, mtDNA copy number and body weight (P ≥ 0.078; Additional file 1: Table S2). Oxidative DNA damage in sperm was not correlated with the levels of courtship and aggressiveness in males that reproduced (courtship: F_1;14_ < 0.01, P = 0.991; aggressiveness: F_1;14_ < 0.01, P = 0.948), but males with a larger relative red area showed a higher level of oxidative DNA damage in sperm (Fig. 1C, F_1;14_ = 8.48, P = 0.011). The level of oxidative DNA damage was not related to any other body state variables measured (P ≥ 0.130, Additional file 1: Table S3).

### Offspring hatching and survival

Mean clutch size (± SD) was 53.207 ± 9.998, mean hatching rate was 89.586 ± 9.878%, and mean survival rate of hatched larvae until age 10 days was 50.528 ± 39.581% (N = 29 families). The hatching success was not repeatable within males, while the survival rate was highly repeatable (hatching: R = 0.011 [0–0.066] 95% CI, P = 0.341; survival: R = 0.610 [0.353–0.757] 95% CI, P < 0.001; overall survival: R = 0.579 [0.315–0.744] 95% CI, P < 0.001; see Additional file 2: Fig. S1).

**Fig. 2:**
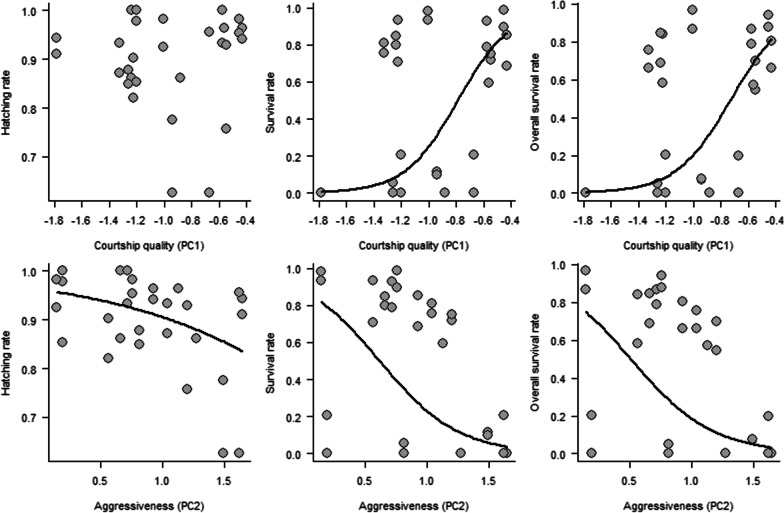
Hatching (first column), survival after hatching (second column), and overall survival considering both hatching and survival rates (third column) of full-sibling families (N = 29) as a function of the courtship quality (first row) and aggressiveness (second row) of fathers expressed during the courtship assay. Aggressiveness and courtship quality are the transformed values. Regression lines are presented for significant relationships (P < 0.050)

Aggressiveness and courtship quality of fathers, which were measured in a courtship behaviour assay, predicted hatching and survival of larvae (Fig. 2). The males displaying aggressive behaviours during the courtship test had reduced offspring hatching and survival rates (hatching: P = 0.005; survival: P = 0.036; overall survival: P = 0.030; Additional file 1: Table S4). The males performing better courtship showed higher offspring survival rates (survival: P = 0.010; overall survival: P = 0.007; Additional file 1: Table S4). There was no statistically significant relationship between relative size of red colour area of fathers and hatching and survival of larvae (P ≥ 0.271; Additional file 1: Table S4). rMMR of father was negatively correlated to hatching rate of offspring (P = 0.050; Additional file 1: Table S4), but other body state traits of males were not related to larvae hatching or survival (P ≥ 0.074; Additional file 1: Table S4).

The principal results are summarised in Additional file 1: Table S5.

## Discussion

In this study, we examined whether secondary sexual traits of male three-spined sticklebacks from a fast-living population are related to their body state traits, as frequently assumed, and tested whether these traits influence the viability of their offspring. Individuals investing in nest construction and courtship behaviour were smaller than those not investing in these reproductive activities. However, there was no evidence that the expression of secondary sexual traits (i.e., red colouration and courtship behaviour) represent the physical and physiological status of males (i.e., metabolic rates, swim ability and mtDNA copy number). Fish investing more in red nuptial colouration showed higher levels of oxidative DNA damage in their sperm, confirming previous evidence that the expression of red colouration trades off against sperm quality in this population (see [29]). Male behaviours during courtship, but not red colouration, were good predictors of offspring viability.

Previous studies of different three-spined stickleback populations have demonstrated positive correlations between secondary sexual traits and male condition. For instance, redder males are relatively heavier to their size [3, 50], have more lipids contents [49] or are less parasitized than duller males [50]. In this study where we explored a variety of physiological and physical measures of male state in an annual stickleback population, we found no evidence that the size of the nuptial area and courtship behaviours of males honestly signal their physiological and physical condition. Although small sample size in this study perhaps limited the statistical power to detect any small effect, these results are in accordance with previous studies using the same population that showed trade-offs between the expression of secondary sexual or other reproductive traits of males and state-related traits either at the phenotypic or at the genetic level. In the study population, redder males carried more oxidative DNA damage in the soma and germline [29], and males simultaneously upregulated metabolic and oxidation–reduction genes and downregulated reproduction-related hormone genes in response to an environmental challenge [53]. The expression of red nuptial colouration has heritable variation in our study population [36], so females may gain indirect benefits if they preferentially mate with a redder male (but see below).

The “good genes” process implies the presence of positive associations between secondary sexual traits and the bearer’s genetic quality [54]. On the other hand, sexual ornaments are costly to produce and maintain [16, 19]. Our results show that the investment of males in red colouration and other reproductive activities incurred oxidative costs. These trade-offs may occur because, for example, sexual signalling based on carotenoids diverts these resources away from other important physiological functions such as antioxidant protection of the soma and germline during growth and reproduction [29, 55]. Carotenoid rich diet appears to increase the quality of parental care in sticklebacks [56], suggesting that red colouration can also compete with this function. These costs may limit the expression of red colouration and reduce the honesty of this signal as an indicator of mate quality [51]. Indeed, in a recent study, we found that the level of oxidative DNA damage in sperm negatively influenced egg fertilization success in the same study population (our unpublished data). It is interesting to note that juveniles from redder families tend to die younger in this population due to genetic conflict between attractiveness and viability [36], and females do not prefer redder males in another adjacent annual population [57]. Nevertheless, red colouration may be selected due to its benefits for male sticklebacks in territory defence against other males [58, 59]. It is important to note that we used relative size of red area as a measure of nuptial colouration, but using different measures of this sexual signal, for example colour intensity, may lead to different results [60–62].

Fitness consequences of sexual traits are often explored to demonstrate the interplay between sexual traits and genetic quality, and positive correlations between sexual ornaments and offspring survival have been found in a wide range of species including both vertebrates and invertebrates [63–68]. However, few studies have considered courtship behaviours as sexual traits to explore their effects on offspring survival [69, but see 70]. Although there was no evidence that courtship quality and aggressiveness of male sticklebacks are correlated with their physiological and physical state, these male behaviours were closely related to the viability of their offspring. Offspring sired by males that perform better courtship and show less aggression toward their mates hatched and survived better, although hatching rate was not repeatable within males. In species where males provide intensive parental care, like the three-spined stickleback, it is difficult to disentangle the effect of genetic quality and parental care on offspring survival [13, 71]. Stickleback males of good quality that show better courtship and less aggression are also likely to provide better care for eggs and larvae and not predate on their offspring [13]. In this study, the fertilised eggs were separated from their fathers then incubated under standardised conditions to remove parental care effect, but offspring survival rate was still highly repeatable within males and correlated with male’s behaviours. Thus, our results demonstrate that aggressiveness and courtship behaviour can signal the genetic quality of male sticklebacks, with strong consequences for offspring viability, which are not attributed to parental care.

Our results suggest that both courtship quality and aggressiveness of males during the courtship are reliable cues of their genetic quality. Thus, females that choose mates based on males’ courtship behaviours might have high fitness. Indeed, aggressive males have a lower probability to successfully mate with a receptive female in the three-spined stickleback [47]. It is interesting to note that our study population located at the southern edge of the species’ European distribution presents a fast pace-of-life. In this annual population, fish go through a single long reproductive season during which females spawn on average six clutches [72], and successful males simultaneously take care of multiple clutches from different females in their nests [73]. Thus, males’ behaviours during courtship, which are related to their cognitive ability [57] and possibly to their parental care behaviour, may be under strong sexual selection in this population. Contrary to expectations, in these annual populations, sexual ornaments of males may not honestly signal their physiological and physical state because they invest at maximum in the single reproductive season due to their relatively low life expectancy compared to northern populations [13, 74].

## Conclusions

We show that sexual signals of male sticklebacks were not a good indicator of their body state, but their aggressiveness predicted offspring viability. In the study population, which represents a fast pace-of-life with high reproductive rate and short lifespan, individuals may invest all their resources in a single reproductive season despite high costs, and thus sexual colouration may not honestly signal their state. Our results suggest that courtship behaviour of a male is a good indicator of its genetic quality that influences offspring viability. Thus, in our study population, we expect that females will increase fitness by choosing males performing good courtship instead of those investing heavily in sexual colouration.

## Methods

### Study population and maintenance

On 10th March 2020, we collected wild adult three-spined sticklebacks with hand nets in the Rio Sar (Galicia, Spain). The fish were brought to the lab and maintained individually in 8-L tanks randomly distributed in three closed flow-through water systems. In these three systems, water was filtered and aerated by mechanical filters and circulation pumps. Visual contact between fish was prevented by opaque lateral walls. Flow-through water coolers ensured that the water temperature matched the seasonal water temperature in the sampling site (14 °C). Natural photoperiod was simulated too with programmed lighting (13 h light: 11 h dark cycle). Tanks were enriched with an artificial plant and a shelter made of a PVC pipe (see Additional file 2: Fig. S2). Fish were fed daily on defrosted bloodworms.

We provided 29 male fish housed in individual tanks with nesting materials, a Petri dish filled with sand and a bunch of 50 green polyester threads (Additional file 2: Fig. S2). Once a fish used all the threads for nesting, more threads were provided until the nest was completed (with a visible entrance hall). Females were monitored daily to record gravidity, which is evident from abdomen size and cloaca opening. To stimulate nest constructions, all males were shown a gravid female enclosed within a transparent glass during 5 min three times per week from 25th March, then once a day from 4th May (i.e., during the peak breeding season) until used for reproduction (see below). Each female was presented to a maximum of six fish per day and was not used for two consecutive sessions (i.e., at least 4-day intervals between two presentation sessions).

### Fish reproduction

During the peak breeding season, males who constructed a nest were used for reproduction (20 out of 29 males) with two different mates. Among these, 18 succeed to mate and produced two fertilized clutches between 27th April and 25th May (N = 36 full-sib families). A breeding session began by releasing a fully gravid female (evident from cloaca opening) inside a male’s tank and lasted up to 30 min. The female was removed if one of the pair presented a highly aggressive behaviour toward the other, or if the male did not display any courtship within 5 min. Once a female spawned and the eggs were fertilized by her mate, she was returned to her home tank. One hour after fertilization, the whole clutch was carefully collected from the nest and placed in an incubator. Then, to assess the male’s nuptial colouration, it was photographed on its left side with a digital camera (Nikon D90) under standardized conditions by following a standard method [3, 49]. For this, the male was immobilized within a transparent plastic box filled with water by using a black sponge. The whole process of fish handling took less than 90 s. We calculated the proportion of the fish lateral area coloured in red by analysing the digital image with a homemade program coded in python and using the open-cv library. The periphery of the fish was manually trimmed and we calculated the proportion of pixels included in given hue, saturation and value ranges (Hue: 340–360 and 0–50; Saturation: 100–255; Value: 0–255).

The 36 fertilized clutches were housed in an 65-L incubation tank, which was well-aerated with several air stones, following standard egg husbandry protocol [75]. One day before hatching (7 days after fertilization), each clutch was transferred to a 10-L tank and provided with three drops of industrial liquid food for newly-hatched larvae (NobilFluid Artemia, JBL©). Two days after hatching (day 2), we began to feed the stickleback larvae twice a day with newly hatched *Artemia*. At day 4, we counted the numbers of alive and dead larvae and unhatched eggs to determine hatching success (i.e., the proportion of eggs hatching and producing viable larvae). The survival of larvae was monitored daily until age 10 days, and dead individuals were removed. In the tanks, the water oxygenation was ensured with an air stone until day 5, after which the air stone was replaced by a sponge filter. Unfortunately, accidental mortalities occurred due to a temporal failure of air supply in six tanks, and these six out of 36 full-sib families have been excluded from the data analyses.

### Courtship behaviour

We assessed the quality of courtship behaviour of all males with a nest (N = 20) on 27th May. Prior to this, all males were exposed daily to a gravid female in the same way as above. For each courtship assay, we presented a gravid female enclosed within a glass in the top right corner of the male tank, allowing visual but not physical contacts between fish (see Additional file 2: Fig. S2), and video-recorded the fish for three minutes using a digital camera (Nikon 90D). Each female was used for up to 6 assays. After the courtship assay, males were weighed with a digital balance (precision 0.001 g) and measured with a metal ruler (precision 0.05 cm). The videos were analysed by an experimenter, blind to the fish identity, using the BORIS software [76]. For each focal male we recorded the occurrences of five different behaviours: leading to the nest (when it faces the female and immediately after swims toward his nest or nesting materials), fanning (while facing the nest, it increases the rate of flapping his pectoral fins, simulating egg ventilation), showing nest entrance (when it inserts its head into the nest entrance and performs small “back and forth”), gluing (when it passes over the nest, rubbing its belly, simulating nest building), and aggression (when it dashes violently toward the female with its mouth open).

### Metabolic rate and swimming performance measurements

Metabolic rates and swimming performance were examined between 29th May and 06th June in all males, both with and without a nest (N = 29). We first measured the standard metabolic rate (SMR) of fish by using an intermittent-flow mini-chamber respirometer system (Loligo Systems, Viborg, Denmark), which simultaneously measured the oxygen consumption of four different fish during an 18-h session. We used a standard protocol described in the (Additional file 3: S1). After the SMR session, the four fish were moved from the respirometer chambers to individual waiting tanks (similar to their home tanks) before the measurements of their maximum metabolic rates (MMR) and swimming performances (critical swimming speed, *U*_crit_) in an intermittent-flow swim tunnel respirometer system (Loligo Systems). We used a standard protocol described in the (Additional file 3: S2). After the measurements of SMR, MMR and *U*_crit_, the fish were euthanized with an overdose of benzocaine anaesthetic.

The sacrificed fish (N = 29) were dissected to extract their left testis as well as a part of their dorsal muscle. Testis was cut into small pieces in 50 µl of buffer and gently agitated with a Vortex mixer to separate sperm from the testis tissue, and then the sperm solution was collected. The sperm and muscle samples were kept at – 80 °C until further use.

### Oxidative DNA damage in sperm and mtDNA copy number in muscle

The relative mtDNA copy number in muscle was estimated by calculating the ratio of mtDNA on nuclear DNA by real-time PCR on a StepOnePlus (Applied Biosystems). The details of the analysis are presented in the (Additional file 3, S3). The level of oxidative damage in sperm DNA was estimated by measuring the quantity of 8-hidroxy-2-deoxyguanosine (8-OHdG, an oxidized derivative of deoxyguanosine). The level of oxidative DNA damage was expressed as quantity in picograms of genomic DNA containing 8-OHdG. The details are presented in the (Additional file 3, S4).

### Data analyses

All the following statistical analyses were done with the R software (R version 4.0.3). Five different behaviours recorded during the courtship test in males with a nest were first analysed by a principal component analysis (PCA, N = 20) using the *FactoMineR* package. The first principal component (PC1) explained 74.39% of the variance and regrouped equally four typical courtship behaviours (i.e., leading to the nest, fanning, showing nest entrance, and gluing). These four behaviours appeared to be highly correlated to each other, suggesting that they carried redundant information (Additional file 1: Table S6). The second component (PC2) explained 19.43% of the variance and was composed with 97.06% of the attack behaviour during the courtship assay (Additional file 2: Fig. S3). Thus, in the subsequent analysis, we used PC1 and PC2 (hereafter, “courtship quality” and “aggressiveness”).

Residuals from the regression models, relating SMR and MMR to body mass (measured immediately after metabolic rate measurement), were used as standardized metabolic rates (respectively rSMR and rMMR) in all statistical analyses [77]. rMMR, oxidative DNA damage in sperm, and behaviours (PC1 and PC2) were transformed with the transformTukey function from the package *rcompanion* [78] prior to the analyses to satisfy the linearity and homoscedasticity criteria: transformed rMMR equals − 1 × (rMMR + 1)^−0.1^, transformed oxidative DNA damage in sperm equals – 1 × DNA_dam_^−0.2^, transformed PC2 equals (PC2 + 1)^0.425^, transformed PC1 equals – 1 × (PC1 + 2)^−0.45^.

We tested whether courtship quality and aggressiveness (PC1 and PC2) were related to physiological state (weight, rSMR, rMMR, *U*_crit_, and mtDNA copy number) in males with a nest (N = 20) by using two linear models (LMs), containing all the physiological traits as independent variables. Since red colour area was measured repeatedly (immediately after successful mating, N = 18 fish), the relationships between physiological traits and relative size of red colour area were analysed in a linear mixed effect (LME) model with male identity as a random factor. We analysed the relationships between sexual traits (courtship, aggressiveness and averaged red colour area) and the level of oxidative DNA damage in sperm by using a LM (dependent variable: sperm DNA damage, N = 18). In another LM, the relationships between body state variables (rMMR, rSMR, *U*_crit_, mtDNA copy number and weight) and oxidative DNA damage in sperm were explored (N = 20).

We examined whether hatching rate (proportion of eggs hatching) and survival rate (proportion of hatched larvae surviving to age 10 days) of offspring were repeatable within fathers by using the *rpt* function of the rptR package [79]. The models included the father identity as a grouping factor and the order of reproduction (first or second clutch) as a fixed effect. One family out of 30 full-sib families was excluded in the statistical analyses because it had an extremely low hatching rate (< 10%, an outlier).

We tested whether fathers’ physiological traits influenced offspring hatching, survival to age 10 days and overall survival (proportion of eggs successfully hatching and surviving to age 10 days) using the *glmer* function (lme4, R package) in generalized linear mixed models (GLMMs) with a binomial error distribution and a logit link function. In the models, parents’ identities were included as random effects, father’s weight, oxidative DNA damage in sperm, rSMR, rMMR, *U*_crit_ and mtDNA copy number in muscle as fixed effects without interactions. The effects of males’ secondary sexual traits (courtship quality, aggressiveness and red colour area) on offspring hatching and survival were also analysed in GLMMs, including parental identities as random variables and red colour area, courtship quality and aggressiveness as fixed effects.

## Supplementary Information

**Additional file 1: Table S1.**Results of t tests and Wilcoxon test for the comparisons of body state traits between the males that built a nest and those that did not. **Table S2.** Results of the LMEs (red coloration) and LMs (courtship and aggressiveness) testing for the effects of physiological traits on the secondary sexual traits. **Table S3.** LMs testing the effects of secondary sexual traits on oxidative DNA damage in sperm. **Table S4.** Results of the GLMMs testing the effects of body state and sexual traits of fathers on egg hatching success, and offspring survival. **TableS5.** Summary of the main effects (statistically significant results) found in our analyses. **Table S6.** Coefficient of correlation (r) between different behaviours of males measured during the courtship. Values in bold indicates significant correlation with P < 0.05. The correlations are done between the number of occurrences of each behaviour.

**Additional file 2: Figure S1.** a) Hatching success, b) survival rate after hatching and c) overall hatching and survival rate of different clutches sired by different males. The father identity is indicated in the x-axes; fathers are ordered according to the mean hatching/survival rate. **Figure S2.** A male’s tank during female presentation. (A) Male, (B) gravid female within a glass, (C) artificial plant, (D) PVC shelter, and (E) nest materials (Petri dish filledwith sand, and threads). **Figure S3.** Principal Component Analysis (PCA) of themale’s behaviours during the courtship test. a) PCA graphic of the variables and b) summary of the contributions of the different variables to the two different axis.

**Additional file 3: S1.** Standard metabolic rate estimation. **S2.** Maximum metabolic rate and swimming performance protocols. **S3.** Measurements of mtDNA copy number in muscle. **S4.** Measurement of oxidative DNA damage in sperm.

## Data Availability

The datasets supporting the conclusions of this article are included within the article and its additional files.

## Abbreviations

CI: Confidence interval
DNA: Deoxyribonucleic acid
GLMM: Generalized linear mixed model
LM: Linear model
LME: Linear mixed effect model
MMR: Maximum metabolic rate
mtDNA: Mitochondrial DNA
PCA: Principal component analysis
PCR: Polymerase chain reaction
PVC: Polyvinyl chloride
rMMR: Residual maximum metabolic rate
rSMR: Residual standard metabolic rate
SMR: Standard metabolic rate
*U*_crit_: Critical swimming speed
8-OHdG: 8-Hidroxy-2-deoxyguanosine
