# Supplementary material for: Relationships between male secondary sexual traits, physiological state and offspring viability in the three-spined stickleback
Source: BMC Ecol Evol. 2022 Jan 7;22:4. doi: 10.1186/s12862-021-01958-8 (PMC8742421; doi:10.1186/s12862-021-01958-8)
Supplement: Supplementary file 1 — Additional file 1: Table S1.Results of t tests and Wilcoxon test for the comparisons of body state traits between the males that built a nest and those that did not. Table S2. Results of the LMEs (red coloration) and LMs (courtship and aggressiveness) testing for the effects of physiological traits on the secondary sexual traits. Table S3. LMs testing the effects of secondary sexual traits on oxidative DNA damage in sperm. Table S4. Results of the GLMMs testing the effects of body state and sexual traits of fathers on egg hatching success, and offspring survival. TableS5. Summary of the main effects (statistically significant results) found in our analyses. Table S6. Coefficient of correlation (r) between different behaviours of males measured during the courtship. Values in bold indicates significant correlation with P < 0.05. The correlations are done between the number of occurrences of each behaviour. [file 12862_2021_1958_MOESM1_ESM.pdf]

Relationships between male secondary sexual traits, physiological state and offspring viability in the three-spined stickleback

Violette Chiara, Alberto Velando, Sin-Yeon Kim

SUPPLEMENTARY TABLES

Table S1: Results of *t* tests and Wilcoxon test for the comparisons of body state traits between the males that built a nest and those that did not.

	Means ± SD		t/W	df	P
	With nest	Without nest			
Residual SMR	0.001 ± 0.041	-0.003 ± 0.044	t = -0.258	14.458	0.800
Residual MMR	-0.030 ± 0.431	0.067 ± 0.563	t = 0.402	14.032	0.694
U_{crit}	8.313 ± 3.665	6.556 ± 4.281	W = 69		0.334
DNA damages (pico)	4.269 ± 1.732	3.645 ± 1.270	t = -1.022	14.737	0.323
mtDNA copy number	1.283 ± 0.315	1.234 ± 0.380	t = -0.338	13.207	0.741
Standard length (mm)	41.550 ± 1.512	43.556 ± 1.758	t = 2.964	13.581	0.011
Weight (g)	1.010 ± 0.110	1.101 ± 0.140	t = 1.729	12.708	0.108

The means of untransformed data are presented.

Table S2: Results of the LMEs (red coloration) and LMs (courtship and aggressiveness) testing for the effects of physiological traits on the secondary sexual traits.

Fixed effects \ Dependant variables	Red colour area (N=18 males)			Courtship quality (N=20 males)			Aggressiveness (N=20 males)		
	F	df	P	F	df	P	F	df	P
rSMR	0.417	1;12	0.531	0.061	1;14	0.809	0.527	1;14	0.480
rMMR	0.002	1;12	0.964	3.626	1;14	0.078	0.052	1;14	0.823
U_{crit}	0.417	1;12	0.531	0.015	1;14	0.905	0.191	1;14	0.669
mtDNA copy number	1.884	1;12	0.195	1.010	1;14	0.332	0.022	1;14	0.884
Weight	4.685	1;12	0.051	6.498	1;14	0.023	0.499	1;14	0.492

In the analysis of relative size of red colour area, identity of father was included as a random factor (red coloration was measured each time a father reproduced). Two-way interactions between different variables were explored but removed from the presented models because they were statistically not significant.

Table S3: LMs testing the effects of secondary sexual traits on oxidative DNA damage in sperm.

Fixed effects \ Dependant variable	DNA damage		
	F	df	P
Secondary sexual traits (N=18 males)			
Red colour area	8.478	1;14	0.011
Courtship	<0.001	1;14	0.991
Aggressiveness	0.004	1;14	0.948
Body state and DNA damages (N=20 males)			
rMMR	0.120	1;14	0.735
rSMR	0.525	1;14	0.481
U*crit*	0.021	1;14	0.888
mtDNA copy number	0.283	1;14	0.603
Weight	2.585	1;14	0.130

Two-way interactions between different variables were explored but removed from the presented models because they were statistically not significant.

Table S4: Results of the GLMMs testing the effects of body state and sexual traits of fathers on egg hatching success, and offspring survival.

Dependant variables / Fixed effects	Hatching success		Survival after hatching		Overall survival	
	Estimate ± SE	P	Estimate ± SE	P	Estimate ± SE	P
Secondary sexual traits (N=29 families)						
Red colour area	4.182 ± 3.803	0.271	-3.247 ± 15.281	0.832	-0.848 ± 14.087	0.952
Courtship	0.514 ± 0.444	0.247	5.161 ± 2.005	0.010	4.955 ± 1.851	0.007
Aggressiveness	-0.977 ± 0.344	0.005	-3.154 ± 1.501	0.036	-3.003 ± 1.383	0.030
Body state and DNA damages (N=29 families)						
rMMR	-6.747 ± 3.436	0.050	-25.439 ± 16.837	0.131	-25.192 ± 15.712	0.109
rSMR	3.800 ± 4.649	0.414	-0.495 ± 20.332	0.981	-1.514 ± 18.954	0.936
U*crit*	0.046 ± 0.055	0.404	0.321 ± 0.220	0.144	0.289 ± 0.204	0.157
mtDNA copy number	-0.366 ± 0.779	0.639	0.302 ± 3.327	0.928	0.413 ± 3.103	0.894
Weight	0.384 ± 1.849	0.836	2.118 ± 7.966	0.790	2.451 ± 7.438	0.742
DNA damage	4.206 ± 5.172	0.416	-45.180 ± 25.241	0.074	-40.637 ± 23.613	0.085

Survival after hatching refers to survival to age 10 days of only hatched individuals and overall survival refers to survival the proportion of eggs hatching and surviving to age 10 days. GLMMS were performed with a binomial error distribution and a logit link function. Two-way interactions between different variables were explored but removed from the presented models because they were statistically not significant.

Table S5: Summary of the main effects (statistically significant results) found in our analyses.

Result section	Comparison between males with and without a nest	Relationship between different traits (in fish with a nest)
Secondary sexual traits and physiological states	- Fish with a nest are smaller in size and weight than fish without a nest	- Smaller fish perform better courtship<br>- Larger fish have a larger red colour area<br>- Oxidative DNA damage in sperm increases with the relative size of red colour area
Offspring hatching and survival		- Offspring from aggressive males have lower hatching and survival<br>- Offspring from males performing better courtship have higher survival rates<br>- rMMR of father is negatively correlated with hatching rate of offspring

Table S6: Coefficient of correlation (r) between different behaviours of males measured during the courtship. Values in bold indicates significant correlation with P<0.05. The correlations are done between the number of occurrences of each behaviour.

	fanning	attacks	lead to the nest	gluing	show entrance
fanning	-	-0.14	**0.86**	**0.94**	**0.97**
aggressions	-0.14	-	-0.22	-0.20	-0.10
lead to the nest	**0.86**	-0.22	-	**0.91**	**0.82**
gluing	**0.94**	-0.20	**0.91**	-	**0.86**
show entrance	**0.97**	-0.10	**0.82**	**0.86**	-
