# Supplementary material for: Relationships between male secondary sexual traits, physiological state and offspring viability in the three-spined stickleback
Source: BMC Ecol Evol. 2022 Jan 7;22:4. doi: 10.1186/s12862-021-01958-8 (PMC8742421; doi:10.1186/s12862-021-01958-8)
Supplement: Supplementary file 2 — Additional file 2: Figure S1. a) Hatching success, b) survival rate after hatching and c) overall hatching and survival rate of different clutches sired by different males. The father identity is indicated in the x-axes; fathers are ordered according to the mean hatching/survival rate. Figure S2. A male’s tank during female presentation. (A) Male, (B) gravid female within a glass, (C) artificial plant, (D) PVC shelter, and (E) nest materials (Petri dish filledwith sand, and threads). Figure S3. Principal Component Analysis (PCA) of themale’s behaviours during the courtship test. a) PCA graphic of the variables and b) summary of the contributions of the different variables to the two different axis. [file 12862_2021_1958_MOESM2_ESM.pdf]

Relationships between male secondary sexual traits, physiological state and offspring viability in the three-spined stickleback

Violette Chiara, Alberto Velando, Sin-Yeon Kim

SUPPLEMENTARY FIGURES

Fig. S1

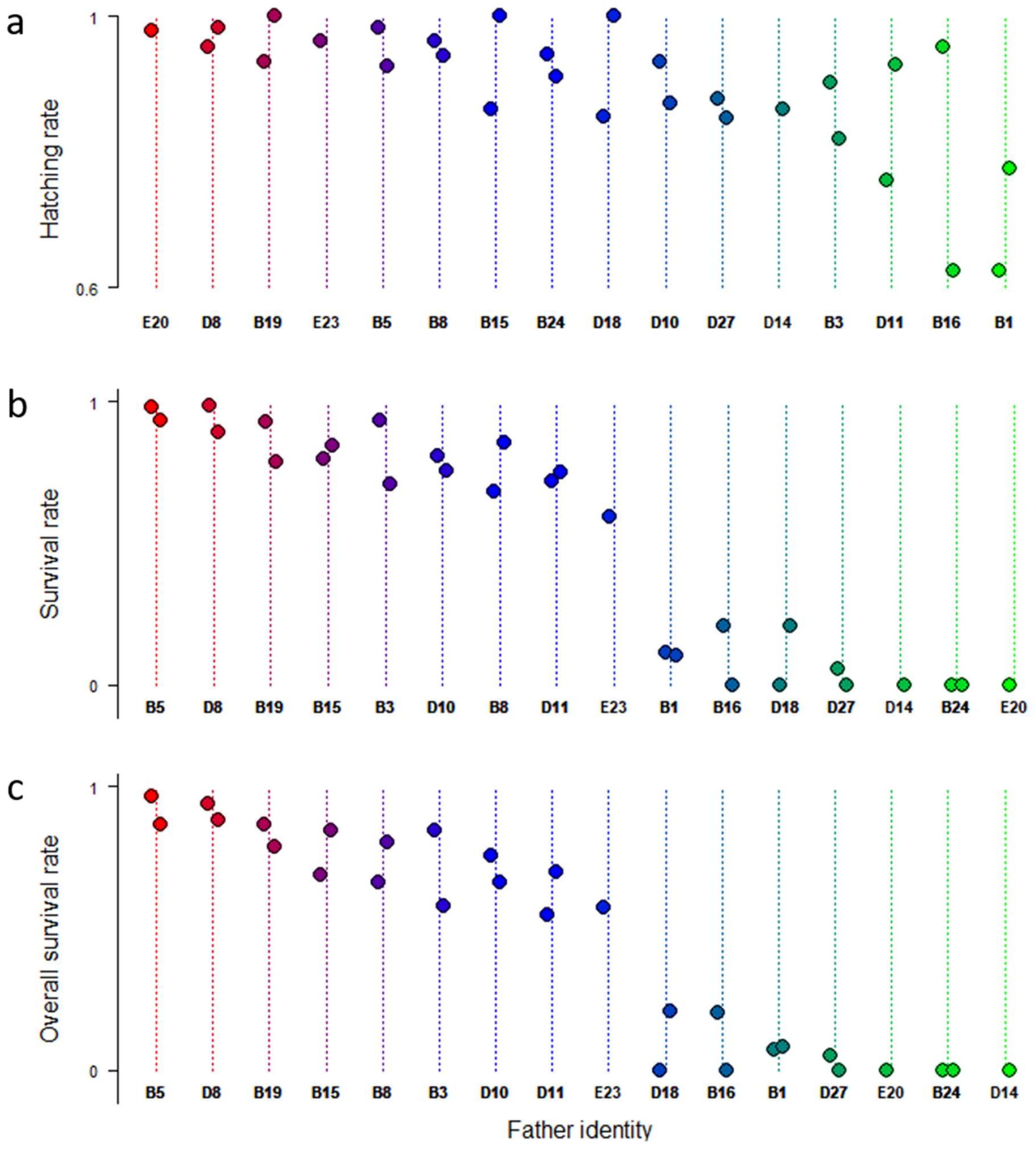

a) Hatching success, b) survival rate after hatching and c) overall hatching and survival rate of different clutches sired by different males. The father identity is indicated in the x-axes; fathers are ordered according to the mean hatching/survival rate.

Fig. S2

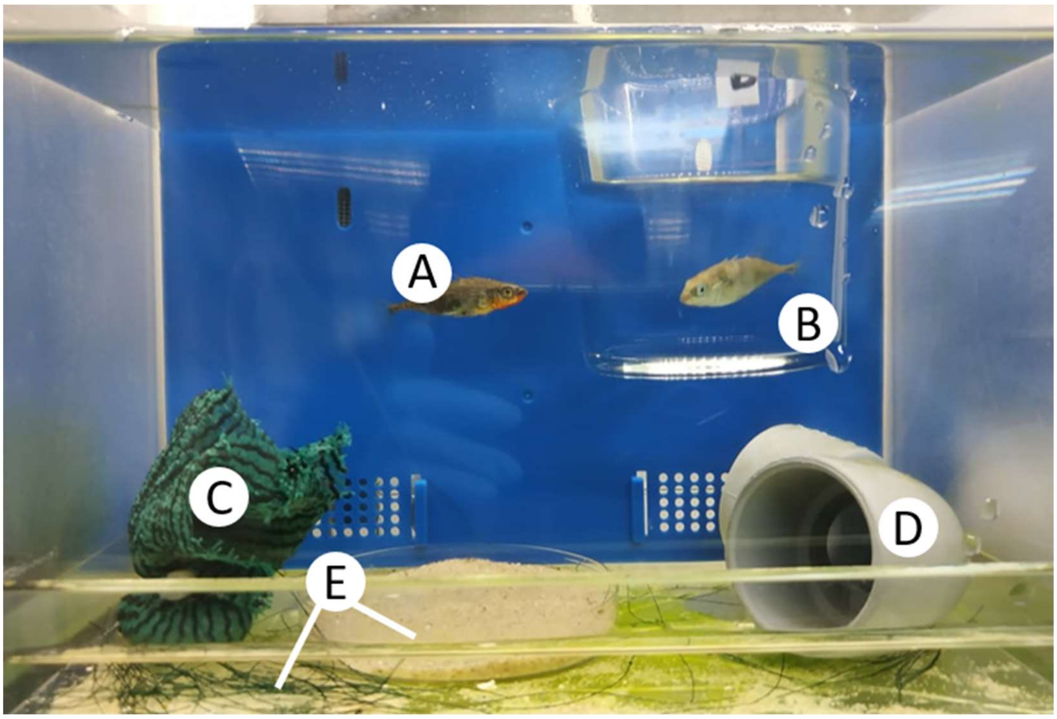

A male's tank during female presentation. (A) Male, (B) gravid female within a glass, (C) artificial plant, (D) PVC shelter, and (E) nest materials (Petri dish filled with sand, and threads).

Fig. S3

a

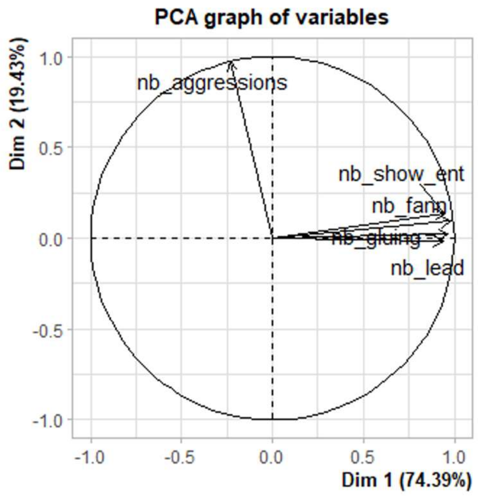

b

	Dim1	Dim2
Fanning	25.73 %	0.91 %
Aggressions	1.45 %	97.06 %
Lead to the nest	23.70 %	0.03 %
Gluing	25.09 %	0.04 %
Showing entrance	24.03 %	1.96 %

Principal Component Analysis (PCA) of the male’s behaviours during the courtship test. a) PCA graphic of the variables and b) summary of the contributions of the different variables to the two different axis.
