# Supplementary material for: Relationships between male secondary sexual traits, physiological state and offspring viability in the three-spined stickleback
Source: BMC Ecol Evol. 2022 Jan 7;22:4. doi: 10.1186/s12862-021-01958-8 (PMC8742421; doi:10.1186/s12862-021-01958-8)
Supplement: Supplementary file 3 — Additional file 3: S1. Standard metabolic rate estimation. S2. Maximum metabolic rate and swimming performance protocols. S3. Measurements of mtDNA copy number in muscle. S4. Measurement of oxidative DNA damage in sperm. [file 12862_2021_1958_MOESM3_ESM.pdf]

Relationships between male secondary sexual traits, physiological state and offspring viability in the three-spined stickleback

Violette Chiara, Alberto Velando, Sin-Yeon Kim

SUPPLEMENTARY METHODS

S1: Standard metabolic rate estimation

We first measured the standard metabolic rate (SMR) of fish by using an intermittent-flow mini-chamber respirometer system (Loligo Systems, Viborg, Denmark), which simultaneously measured the oxygen consumption of four different fish during an 18-hour session. The system consisted of four glass chambers of 13.4 ml and a temperature probe, which were submerged within a 12 L buffer tank. Water temperature in the system was maintained constant (16.47 ± 0.2 ºC) by continuously circulating water between the buffer tank and another 50 L tank equipped with a water cooler and a water pump. Each of the four fish chambers was equipped with an independent fiber optic oxygen sensor, a flush pump, and a recirculation pump. A centralised data acquisition device registered oxygen and temperature data every second. The oxygen sensors were calibrated according to the manufacturer's instructions prior to the use. We programmed 5-min cycles of automatic water flushing and measurements (120 s flushing, 20 s waiting and 160 s measuring) by using the ®AutoResp software (Loligo Systems, Viborg, Denmark).

To prevent an increase in metabolic rate due to digestion, fish were not fed for 24-h prior to the test. In each session of SMR measurement, four randomly selected fish were introduced to the chambers around 3 p.m. and kept undisturbed for a total of 18 hours. The room was kept dim during the day (7h) or completely dark at night (11h). During the whole process, changes in the level of dissolved oxygen were recorded in mg O_2 L^{-1} every second. Oxygen level never dropped below 45.4%. We calculated metabolic rate at each intermittent cycle in mg O_2 h^{-1} with equation 1:

$$\text{Equation 1:} \quad MO_2 = \frac{\Delta O_2(Vt - Vf)}{\Delta t}$$

with ΔO_2 the difference in dissolved oxygen between the first and the last measurement, Δt the duration of the measurement (160-s), and Vt and Vf respectively the tunnel and the fish volumes in litters. We assumed that the fish density was of 1 kg L^{-1} and so that its weight in kg was equivalent to its volume in litters. We obtained the SMR of each individual by calculating the average of the lowest 10% of all $M0_2$ measured during 18 hours.

S2: Maximum metabolic rate and swimming performance protocols

Maximum metabolic rate (MMR) and swimming performance (critical swimming speed, U_{crit}) of fish were determined by using an intermittent-flow swim tunnel respirometer system (Loligo Systems, Viborg, Denmark) immediately after the 18-hour SMR measurement session. The respirometer system

consisted of a 170 ml swim tunnel submerged inside a 20 L buffer tank, a flush pump, a fiber optic oxygen sensor, a temperature probe, and a variable-voltage motor, controlling the water flow inside the swim tunnel. Water temperature in the respirometer was maintained constant in the same way as in the mini chamber system. The flush-measurement cycle was the same as in the SMR session (120 s flushing, 20 s waiting and 160 s measurement).

In each individual session, a fish was placed inside the swim tunnel with the inital water flow of 2.1 cm s^{-1} (approximately 0.5 body length s^{-1}) and kept undisturbed for 20 minutes. After this acclimation period, the flow rate was increased by 2.1 cm s^{-1} every five minutes until the fish was too exhausted to keep his position in the tunnel (position lost for > 30 uninterrupted seconds). We recorded the water velocity and time when the fish became fatigued. After this exercise, the fish remained in the swim tunnel at a low water flow (1.5 cm s^{-1}) during 30 minutes to determine his MMR during this recovery time. The MO_2 at each cycle was calculated with Equation 1, and we used the highest observed value of MO_2 as the MMR of the fish. U_{crit} was determined following a standard method (Brett, 1964; Kolok, 1999) with Equation 2:

Equation 2: $U_{crit} = U_f + U_s \frac{t_f}{t_s}$

with U_f the highest flow rate (cm s^{-1}) maintained for an entire interval (t_s = 5 min), U_s the flow increment between intervals (2.1 cm s^{-1}), and t_f the time until exhaustion in the last interval. Oxygen level never dropped below 69.3%.

S3: Measurements of mtDNA copy number in muscle

We estimated the relative mitochondrial DNA (mtDNA) copy number by calculating the ratio of mtDNA on nuclear DNA by real-time PCR on a StepOnePlus (Applied Biosystems). We used a commercial kit (Quick-DNA Miniprep Plus Kit, Zymo Corp) and followed the instruction to extract DNA from the muscle samples.

We used Glyceraldehyde-3-phosphate dehydrogenase (GAPDH) as a single copy region in the nuclear genome, with direct primer (GAPDH-F), 5'- GAGACGTGACCATTGAGGGG-3'; reverse primer (GAPDH-R), 5'- TGTGCGGGTGGGCTTTATGAT-3'. The cytochrome oxidase subunit 1 (CO1) gene was used as mitochondrial gene. We choose primer sequences designed to amplify a fragment of CO1 gene (99bp) using the sequence from Ensembl (ENSGACT00000027727, BROAD S1, http://www.ensembl.org/Gasterosteus_aculeatus/Info/Index,). For the CO1 gene, the primer sequences were: forward primer (CO1-F), 5'- GGAGGCTTTGGCAACTGACT-3'; reverse primer (CO1-R), 5'-AGAGGGTGGGAGCAATCAGA-3'. We used the BLAST tool (NCBI) search for *Gasterosteous aculeatus* to search the primers and sequences, which confirmed sequence identity and the specificity of primers for the COI sequences. These searches did not show significant result in nuclear genes. Electrophoresis analyses and melt curve also comfort us that only one amplicon was generated by PCR with the chosen COI primers.

For the real-time PCR assay, we performed reactions in a volume of 25 µl including 5 µl of template DNA diluted at 2 ng/uL, primers at final concentration of 200nM and 12.5 µl Luminaris Color HiGreen High ROX qPCR Master Mix (Thermo Scientific). 96-well plates were used to perform COI reactions; the qPCR conditions were 95ºC during 10 min, then 40 cycles of 15 s at 95ºC and 60s at 60ºC.

All samples were run in triplicate on the same plate, one individual sample was used as a reference. Quantification cycle (*Cq*) values appeared to be highly repeatable in the triplicates ($R = 1$, $P < 0.001$). The reference sample was used to estimate the within plate variation. The mean intra-assay variations (CV) of the *Cq* values were <1%. We used the LinRegPCR software (Ruijter et al. 2009) to estimate the amplicon efficiency from the slopes of the amplification curves for each qPCR reaction and averaged for each gene. The mean reaction efficiencies were 1.8683 for COI and 1.8804 for GAPDH. The relative mtDNA copy number (arbitrary units) of each sample was calculated as $Ef^{\Delta Cq}$. With *Ef* the amplicon efficiency, ΔCq the difference in *Cq*-values between the reference sample and the focal sample. We used the mean *Cq* values of the triplicates in all calculations.

S4: Measurement of oxidative DNA damage in sperm

For the sperm DNA damages analysis, each sample was centrifuged and the supernatant with the sperm cells was collected. We then extracted DNA from sperm using a commercial kit (Quick-DNA Miniprep Plus Kit, Zymo Corp.). In 200 ng of DNA, we measured the quantity of 8-hydroxy-2-deoxyguanosine, 8-OHdG (an oxidized derivative of deoxyguanosine) using a commercial kit (EpiQuik™ 8-OHdG DNA damage Quantification Direct Kit, Epigentek Group Inc) and following the manufacturer's instruction (Kim et al. 2019; Kim and Velando 2020). 8-OHdG represents the most abundant pre-mutagenic lesions in DNA and is a direct measure of oxidative DNA damage in the tissues (Valavanidis et al. 2009). To detect the 8-OHdG present in sperm DNA, we used detection and capture antibodies (highly specific to 8-OHdG without cross-reactivity to other 8-OHdG analogues; e.g. dG, guanine, 8-OHGua and 8-OHG).

The signal was enhanced with an enhancer solution followed by the reading of the absorbance using a spectrophotometer at 450 nm (Synergy™ 2 Multi-Mode Microplate Reader, Bio-Tek Instruments Inc.). We calibrated samples with the 8-OHdG standard. The assays showed high repeatability ($R = 0.914$, $P < 0.001$). The level of oxidative DNA damage was expressed as quantity in picograms of genomic DNA containing 8-OHdG.

REFERENCES:

J. R. Brett. (1964) The Respiratory Metabolism and Swimming Performance of Young Sockeye Salmon. *Journal of the Fisheries Research Board of Canada*. **21**(5): 1183-1226.

A S Kolok. (2011) Interindividual variation in the prolonged locomotor performance of ectothermic vertebrates: a comparison of fish and herpetofaunal methodologies and a brief review of the recent fish literature. *Canadian Journal of Fisheries and Aquatic Sciences*. **56**(4): 700-710.

J. M. Ruijter, C. Ramakers, W. M. H. Hoogaars, Y. Karlen, O. Bakker, M. J. B. van den Hoff, A. F. M. Moorman. (2009) Amplification efficiency: linking baseline and bias in the analysis of quantitative PCR data. *Nucleic Acids Research*. **37**(6): 45.
